# Increased Extracellular Matrix Protein Production in Chronic Diabetic Complications: Implications of Non-Coding RNAs

**DOI:** 10.3390/ncrna5010030

**Published:** 2019-03-22

**Authors:** Saumik Biswas, Subrata Chakrabarti

**Affiliations:** Department of Pathology and Laboratory Medicine, Western University, London, ON N6A5A5, Canada; sbiswas7@uwo.ca

**Keywords:** diabetic complications, epigenetics, lncRNAs, histone modifications, miRNAs, ECM alterations

## Abstract

Management of chronic diabetic complications remains a major medical challenge worldwide. One of the characteristic features of all chronic diabetic complications is augmented production of extracellular matrix (ECM) proteins. Such ECM proteins are deposited in all tissues affected by chronic complications, ultimately causing organ damage and dysfunction. A contributing factor to this pathogenetic process is glucose-induced endothelial damage, which involves phenotypic transformation of endothelial cells (ECs). This phenotypic transition of ECs, from a quiescent state to an activated dysfunctional state, can be mediated through alterations in the synthesis of cellular proteins. In this review, we discussed the roles of non-coding RNAs, specifically microRNAs (miRNAs) and long non-coding RNAs (lncRNAs), in such processes. We further outlined other epigenetic mechanisms regulating the biogenesis and/or function of non-coding RNAs. Overall, we believe that better understanding of such molecular processes may lead to the development of novel biomarkers and therapeutic strategies in the future.

## 1. Introduction

Chronic diabetic complications remain a major cause of morbidity and mortality in the diabetic population. Recent estimates by the International Diabetes Federation have suggested that over 693 million people worldwide will be affected by diabetes in 2045, which further implies that diabetic complications will impose an enormous global health problem in the near future [1]. Presently, the prevalence of diabetes is particularly high among certain populations: the elderly; aboriginals; and in immigrants from Hispanic, Asian, and African backgrounds [2,3]. In general, diabetics are at a markedly increased risk for developing long-term vascular complications that affect various organs and contribute to subsequent suffering [4,5].

When considering the detrimental biological consequences of diabetes, the heightened production of extracellular matrix (ECM) proteins is a characteristic feature of all chronic diabetic complications [6,7]. Depending on the unique architectural features of a particular organ, such structural changes in the ECM may manifest in an organ-specific way. For example, in the renal glomerulus, we observe mesangial matrix expansion, tubulointerstitial fibrosis, nodular glomerulosclerosis (evidenced by Kimmelstiel–Wilson nodules), and capillary basement thickening [8]; whereas in the retina, ECM alterations are manifested only as capillary basement membrane (BM) thickening [9]. In the myocardium, these biosynthetic changes in the ECM present as interstitial and perivascular fibrosis [10]. Collectively, the uncontrolled production of ECM proteins ultimately leads to significant deficiency of organ function. 

Although multiple cells may contribute to the increased production of ECM proteins in a tissue-specific manner, glucose-induced endothelial damage may be a key factor in the pathogenesis of such processes [6,7,11]. Given their location in the blood vessel, endothelial cells (ECs) are the first cell types that are exposed to systemic hyperglycemia. Furthermore, in tissues like the retina, glucose uptake in ECs is insulin-independent [12]. Therefore, increased intracellular glucose triggers a number of metabolic disturbances that initially originate in the ECs [7]. 

In order to adapt to the changes in the cellular environment, the transcriptional machinery in ECs is continuously stimulated and modified, which results in the activation of several molecular pathways involved in the aberrant production of ECM proteins—ultimately leading to phenotypic alterations of the ECs [6,7,11,13]. Non-coding RNAs (ncRNAs), which are key players in epigenetic regulation, can significantly influence such biosynthetic processes. In this review, we will specifically address the significance and mechanisms of augmented ECM protein production in chronic diabetic complications, and then discuss the mechanistic roles of these ncRNAs.

## 2. ECM—The Footprint of Chronic Diabetic Complications

In the blood vessels, the ECM provides a scaffold for cellular organization. However, beyond the role of structural support, the ECM can regulate cellular proliferation, migration, and stabilization [14]. The ECM can also control important cellular events such as cell survival, growth, and apoptosis through the binding of endothelial integrins [15]. Of note, growth factor-induced cellular changes are, to some extent, ECM-dependent [16,17]. 

Increased expressions of specific ECM proteins, such as fibronectin (FN), collagen, and tenascin-C, along with reduced productions of laminin and collagen (IV), have been documented during tumor progression [18,19,20]. It is also possible that such changes in the ECM stimulate angiogenesis [21]. This notion is further supported by the fact that similar alterations have been demonstrated during retinal vascular development [22,23]. Notably, retinal tissues show increased FN expression during vascularization, whereas laminin expression is only seen during vascular maturation [18,19]. Moreover, following the onset of diabetes, retinal BMs in diabetic animals have been shown to contain early increases in collagen, laminin, tenascin, and FN [24]. Other organs, such as the heart and kidneys, also show a similar profile of ECM proteins in diabetes [25,26]. 

ECM proteins can also display distinctive functional properties through alternative splicing, or proteolysis, during specific biological or disease processes. For instance, although a single FN transcript can be alternatively spliced into several variants (~20 variants) [27], diabetic environments can evoke the formation of particular FN isoforms (extra domain-B; ED-B^+^ FN) that can further promote the proliferation and differentiation of ECs [28,29]. Similarly, we have demonstrated increased levels of ED-B^+^/oncofetal FN in the vitreous humors of patients with proliferative diabetic retinopathy and in the retinas of diabetic rats [28]. In accordance with our findings, other studies have also determined the biological significance of FN and its fragments on EC function [30,31]. Collectively, these findings indicate that the components of the ECM are organized in a highly regulated manner and any aberrations in this process can ultimately govern ECM architecture and cellular function.

Furthermore, in contrast to the retina, diabetes can evoke anti-angiogenic mechanisms in the heart [32]. The exact reason for such a differential endothelial activation is not completely understood. However, the distinct cellular constituents and the biological functions of the organs may influence the molecular response to a particular disease. For example, as insulin signalling plays a critical role in cardiac metabolism, insulin may be capable of inducing vascular endothelial growth factor (VEGF) expressions (through PI3K/Akt signalling) in various cells that comprise the cardiac tissues—fibroblasts, ECs, and cardiomyocytes [33]. During diabetes, however, certain anomalies in the metabolic pathways (i.e., prolonged activation and production of advanced glycation end-products and their receptors; AGE/RAGE system) may contribute to insulin resistance, which could ultimately impact the ECM architecture and reduce the insulin-stimulated VEGF expressions in the heart; thereby, possibly explaining the observed anti-angiogenic phenotype [33,34,35]. Nevertheless, with tissue-specific responses existing in a diabetic environment, exploring the molecular crosstalk between different cell types becomes paramount.

## 3. Cellular Phenotypic Changes Causing Increased ECM Protein Productions in Chronic Diabetic Complications

As a result of sustained hyperglycemia and altered transcriptional mechanisms (please see the section below), some changes occur in cellular phenotypes that further exacerbate the production of ECM components. For example, following the continuous stress induced by chronic diabetes, particular subpopulations of native fibroblasts in certain organs may show myofibroblastic transdifferentiation that can provoke ECM protein production, organ remodeling, and fibrosis [36,37]. Similarly, during diabetes, tubular epithelial cells in the kidneys may undergo a similar mechanism, known as epithelial-to-mesenchymal transition (EMT), mediated through TGF-β, which can negatively impact the molecular composition of the ECM [38]. Alternatively, the endothelium can also lose its inherent physiological properties and develop a mesenchymal-like phenotype during chronic hyperglycemia [39,40], which is known as endothelial-to-mesenchymal transition (EndMT). EndMT may be one of the mechanisms contributing to the heightened production of ECM proteins in diabetes, especially in tissues lacking fibroblasts (e.g., retina) [40]. Recently, EndMT and associated vascular instability have been hypothesized to be a unifying concept of multiple vascular diseases, including diabetic complications [41]. Nevertheless, alterations in gene transcription are fundamental processes central to all such cellular phenotypic transformations.

## 4. Hyperglycemia and Gene Transcription

In ECs, hyperglycemia causes oxidant injury as a result of glucose autoxidation, AGE/RAGE interactions, alterations of NADPH/NADP^+^ and NADH/NAD^+^ ratios, depletion of antioxidants and cofactors, and mitochondrial NADPH oxidase activation [7,42,43,44]. Increased glucose flux through the electron transport chain and augmented O_2_^−^ production potentiate oxidant injury [7,45,46,47]. We have previously demonstrated that in diabetes, oxidative stress causes the activation of redox-sensitive transcription factors (please see Section 4.1.1.), which can heighten the production of multiple inflammatory mediators [48,49,50]. In addition to metabolic stress mechanisms, transcriptional co-activators with histone acetyltransferase (HAT) activity, such as p300, can also regulate nuclear factor kappa B (NF-κB) and several transcription factors [49,51] (please see Section 4.1.2.).

### 4.1. Transcription Factors and Co-Activators

#### 4.1.1. NF-κB and AP-1

In order to meet the cellular demands of prolonged hyperglycemia, a plethora of DNA-binding proteins (known as transcription factors) are activated and recruited to the promoter regions of transcriptionally active genes. Among the diverse groups of transcription factors, nuclear factor kappa B (NF-κB) is one transcription factor that is practically found in all cell types and plays a central role in the progression of diabetes [52,53]. Notably, several stimuli such as hyperglycemia, oxidative stress, and AGEs can trigger the activation of NF-κB by phosphorylating and degrading its inhibitory proteins, IκB, in the cytoplasm [48,52,53,54]. Following subsequent activation and translocation into the nucleus, the NF-κB dimers (generally comprised of p65 and p50 subunits) can then enhance the transcription of critical genes that encode for various inflammatory mediators and ECM proteins [49,50,51,52,53,54,55,56]. 

In addition to NF-κB, activator protein 1 (AP-1) is another prominent family of transcription factors that can play an important role in cell survival, proliferation, inflammation, and cell cycle regulation [57,58]. AP-1 dimers are comprised of Jun, Fos, activating transcription factor (ATF), and Jun dimerization protein (JDP) subunits that share a basic leucine zipper (bZIP) domain [57,58,59]. Depending on the subunits present, certain dimerization motifs of AP-1 can determine the degree of transcriptional activity and DNA-binding affinities; the most prevalent and stable form of AP-1 exists as the Jun–Fos heterodimer [57]. Furthermore, a large variety of stimuli can induce the activity of *AP-1* genes (i.e., *Fos*) through signalling cascades, such as MAPK/ERK, that coordinate the phosphorylation of certain transcription factors (i.e., ternary complex factors) in the nucleus, furthering the formation of AP-1 dimers [57,59].

Although the regulation of AP-1 and NF-κB exhibits distinct signalling pathways, significant crosstalk can occur between these two transcription factors following certain stimuli [60]. For example, we have previously reported that hyperglycemia can heighten the activation of both NF-κB and AP-1, which then increases the production of ECM proteins and vasoactive factors in ECs and in cardiac, kidney, and retinal tissues affected by chronic diabetes [48,49]. In parallel with our findings, other reports have documented similar patterns between NF-κB and AP-1 in certain cells during hyperglycemia/diabetes—vascular smooth muscle cells [61] and peripheral blood mononuclear cells [62]. Of note, AP-1 and NF-κB are merely two examples of a vast array of transcription factors that exist. Several transcription factors such as cAMP-response element-binding protein (CREB) [63], nuclear factor of activated T cells (NFAT) [64], stimulating protein 1 (SP-1) [65], signal transducer and activator of transcription (STAT) [66], forkhead box O (FOXO) [67], and early growth response gene-1 (Egr-1) [68] also have critical implications in gene regulation during disease pathogenesis. Consequently, future research must continue to examine the molecular networks in its entirety, as novel interacting partners may be discovered using such an approach. After all, ncRNAs [69,70] and other epigenetic mediators, such as p300 (please see Section 4.1.2.), can control the expressions of various transcription factors [49,54], providing an added layer of complexity to transcriptional regulation.

#### 4.1.2. p300 and Histone Acetylation

In the chromosome, DNA is tightly packaged around histone proteins. At the time of transcription, acetylation of particular amino acid residues in histones, carried out by histone acetyltransferases (HATs), unpacks the DNA, making it accessible to transcription factors. On the other hand, histone deacetylation, which is mediated by histone deacetyltransferases (HDACs), is typically associated with gene silencing. Such post-translational modifications of histones are one of the major contributors of gene regulation at the chromosomal level [71,72,73,74]. In fact, the transcriptional co-activator p300 is the most well-known histone acetylator and regulates several cellular processes via multiple transcription factors such as AP-1 and NF-κB [74,75,76]. p300 plays a crucial role in differentiation and growth [74] and we have also shown that p300 is upregulated in the retinal and cardiac tissues of diabetic animals [49,51,55]. Interestingly, p300 is further capable of influencing the production of long non-coding RNAs (lncRNAs) and microRNAs (miRNAs) [40,55,77,78,79,80,81], suggesting that such chromatin-modifying mechanisms are pivotal in gene regulation.

### 4.2. Non-Coding RNAs

Several classes of ncRNAs (e.g., miRNA, lncRNA, snoRNA, circRNA, and piwi-interacting RNA) have been described. However, in view of the tremendous amount of ncRNAs known today, this review will only focus on some of the well-established miRNAs and lncRNAs that are shown to impact ECM proteins in diabetes-related complications.

#### 4.2.1. Non-Coding RNAs

In recent years, the discovery of RNA-based gene regulation has expanded our understanding in all biological processes. miRNAs, a group of small ncRNA molecules (~20–25 nucleotides in length), are one of the major players in such processes [82,83]. Similar to mRNAs, miRNAs are produced in the nucleus by RNA polymerase II. However, after transcription, primary miRNAs are subsequently processed into precursor (pre-) miRNAs by a multiprotein complex that is comprised of Drosha (a RNase III enzyme) and DiGeorge Syndrome Critical Region 8 (DGCR8; an RNA-binding protein). Following processing, a protein known as Exportin 5 then shuttles the pre-miRNAs to the cytoplasm, where they are further processed and assembled into mature, functional miRNAs by Dicer (another type of RNase III) and RNA-induced silencing complex (RISC), respectively. From there, the miRNAs act as negative regulators by degrading and/or translationally inhibiting their target mRNAs [82,83,84]. Furthermore, miRNAs can also play important roles in regulating histone modifications [85,86,87] and certain lncRNAs [88,89,90]; while paradoxically, lncRNAs can regulate both miRNAs and histone remodeling complexes [91,92,93,94] (please see Section 7). 

#### 4.2.2. Long Non-Coding RNAs (lncRNAs) 

The vast span of non-coding regions in the genome brings us to question the nature of its role in the cellular system—especially the poorly understood lncRNAs. The idea of lncRNAs first surfaced nearly three decades ago with the description of X-inactive specific transcript (*XIST*), the lncRNA that is responsible for X-chromosome inactivation in eutherian lineage [95,96]. LncRNAs are defined as transcripts greater than 200 nucleotides that do not have protein-coding potential and may be transcribed by RNA polymerase II or III, are subject to splicing, and may even be composed of a single exon [97]. They were once considered the ‘dark matter of genome’ and unlike protein-coding genes, lncRNAs generally demonstrate poor sequence conservation and lack open reading frames. Following transcription, lncRNAs can regulate target genes via the cis effect (on neighboring genes on the same chromosome) or trans effect (on distant genes on the same or other chromosomes) through transcription factors or RNA polymerases. On the basis of their location and orientation relative to nearby genes, lncRNAs can be classified as sense/antisense, divergent/convergent, or intronic/intergenic [92,98]. Moreover, lncRNAs can employ various mechanisms to influence the expression of their target genes: (a) promote signalling mechanisms by directly regulating gene expression and activity, (b) act as a decoy to prevent the attachment of transcription factors to specific promoter regions, (c) act as a guide to orient proteins to locations where they can promote further protein modifications and alter gene expressions, and d) serve as a scaffold to assist with the assembly of pertinent molecular units. LncRNAs play roles in embryogenesis and in several diseases such as cancer and neurodegeneration [99,100,101]. These tissue-specific RNAs tend to also form thermodynamically stable secondary and higher-order structures. Furthermore, the myriad functional roles of lncRNAs include modulation of alternative splicing, chromatin remodeling, cis/trans-acting regulators of gene expression, cytoplasmic gene regulation, and RNA metabolism [102,103,104,105,106,107].

## 5. Role of miRNAs in Chronic Diabetic Complications in Various Organs

Under certain disease contexts, miRNAs can exhibit either protective or damaging effects by post-transcriptionally regulating mRNAs. As outlined in Table 1, we and others have shown that several miRNAs are altered in chronic diabetic complications and can consequently regulate the expressions of ECM proteins and angiogenic factors via distinct signalling cascades—ultimately contributing to glucose-induced oxidative stress and DNA damage, which lead to structural and functional deficits in cells [7,11,47]. We will discuss the involvement of miRNAs in such processes in the sections below. Of note, we do recognize that there are additional uncharacterized miRNAs that may be of further importance.

### 5.1. miRNAs in Diabetic Nephropathy (DN)

Impaired renal functions, along with glomerulosclerosis, are characteristic features of diabetic nephropathy (DN). At the protein level, glomerulosclerosis occurs as a result of the increased production and deposition of ECM proteins, including collagen and FN. Kato et al. have demonstrated that such increases in ECM proteins (i.e., collagen 1α2) are attributed to elevated TGF-β1 signalling, which is also associated with miR-192 upregulation in the renal glomeruli of type 1 and type 2 diabetic animals [108]. In accordance with Kato et al., Deshpande and colleagues reported a similar phenotype for miR-192, in which the pathological features of DN (kidney fibrosis, proteinuria, albuminuria, and hypertrophy) were attenuated in the *miR-192* knockout (KO) diabetic mice [109]. Interestingly, both research groups postulate that TGF-β1 signalling may be self-sustained by the activation of feedback loops that promote crosstalk between miRNAs (miR-192 and miR-200b/c) and various transcription factors (p53, Zeb1/2) implicated in the pathogenesis of DN [109,110]. Conversely, Krupa et al. observed reduced levels of miR-192 in the renal tissues of patients with severe DN (at stage 5 chronic kidney disease), whereas patients with early DN features had slightly elevated miR-192 levels [111]. Using additional in vitro experiments, Krupa et al. indicated that TGF-β1 treatments can downregulate miR-192 and E-cadherin in proximal tubule epithelial cells, while overexpressing miR-192 enhanced E-cadherin levels—suggesting a possible protective role for miR-192 [111]. Similarly, in a study by Wang et al., diabetes was also found to decrease both miR-192 and miR-215 levels in the renal cortex of *apoE* KO animals [112]. The investigators additionally demonstrated in vitro that miR-192/215 are capable of targeting zinc finger E-box binding homeobox2 (ZEB2), which is a prominent transcription factor that can negatively regulate the expressions of several genes including *E-cadherin* [112]. Nevertheless, these findings suggest that differential effects of the same miRNA may exist during various stages of disease development. It is also possible that the application of different cell and animal models may contribute to the contrasting miRNA patterns.

Moreover, miR-377 has been shown to contribute to the pathogenesis of DN by indirectly upregulating FN through the suppression of p21-activated kinase and superoxide dismutase, which are upstream inhibitors of FN [113]. In contrast to the pathogenetic capabilities of miR-377, Wang et al. recently demonstrated that miR-let-7b has a protective role against diabetes-induced renal fibrosis through an inhibitory effect on the TGF-β1 receptor and its downstream signalling components [114]. Therefore, on the basis of their findings, miR let-7b was proposed as a potential therapeutic target for renal fibrosis in DN [114]. Similarly, in an interesting study by Long et al., hyperglycemia is capable of downregulating the promoter activity of minichromosome maintenance complex 7 (MCM7) to ultimately reduce miR-93 (another protective miRNA), which normally inhibits excessive VEGF-A expressions along with collagen and FN deposition in podocytes [115]. Hyperglycemia-induced TGF-β1 signalling can also repress the expressions of miR-29 family members (miR-29a, miR-29b, and miR-29c), which subsequently promotes kidney fibrosis through the heightened production of collagen [116,117]. However, contrary to the protective functions of miR-29, Long et al. have additionally suggested that miR-29c can be a detrimental miRNA, because of its involvement in renal fibrosis, by inhibiting sprouty homologue (SPRY1) protein and upregulating Rho Kinase signalling [118]. More generally, elevated miR-29c is able to increase the deposition of ECM proteins (i.e., FN) in renal mesangial cells of diabetic mice and inhibition of this miRNA can further attenuate mesangial matrix accumulation and albuminuria [118].

Phenotypic alterations of certain cells in the diabetic kidneys, possibly through EMT, may be one of the major contributing pathogenetic factors in renal fibrosis. For example, Loeffler et al. demonstrate that the genetic ablation of *Col8α1/α2* (an important gene that regulates collagen VIII expressions) can reduce the amount of interstitial myofibroblasts and EMT-like alterations in the diabetic mice kidneys [119]. Interestingly, significant reductions in ECM protein productions, kidney fibrosis, and albuminuria were also observed in the *Col8α1/α2* KO diabetic mice kidneys compared to diabetic wild-type (WT) animals. Furthermore, during diabetes, members of the miR-200 family (miR-200b/c) can also influence the composition of glomerular mesangial cells by directly inhibiting zinc finger E-box binding homeobox (ZEB) proteins, subsequently contributing to heightened production and deposition of various ECM proteins (Col1a2, Col4a1) [110]. In addition to these findings, Park et al. show that miR-200b/c can inhibit FOG2, a PI3K inhibitor, which in turn allows for greater TGF-β-induced Akt activation, leading to hypertrophy of mouse glomerular mesangial cells in DN [120]. As for miR-200a, Wei et al. indicated that aldose reductase (a polyol pathway enzyme; AR) is primarily responsible for the negative regulation of miR-200a-3p in DN [121]. Using the renal cortical tissues from *Ar* KO diabetic mice, Wei et al. observed that elevated miR-200a-3p levels were associated with reduced oxidative damage and TGF-β-induced fibrosis and EMT; these patterns were also confirmed by in vitro experiments. In parallel, shRNA-mediated knockdown of miR-200a-3p in the diabetic animals provoked fibrogenesis in the glomerular and cortical tissues. In general, miRNAs are implicated in a complex network that can impact diverse signalling pathways during disease pathogenesis.

Nevertheless, several miRNAs are known to influence FN synthesis, which include miR-21, miR-146a, and miR-1207-5b [55,122,123,124]. More specifically, miR-21 has been shown to enhance FN deposition [125] and promote fibrosis in kidneys with the involvement of TORC1 (transducer of regulated cAMP response element binding activity 1) proteins [123]. Downregulation of miR-21 imparts protection against this situation [123]. In contrast, miR-146a is downregulated in the kidneys during diabetes. Endothelial-specific miR-146a overexpression can prevent diabetes-induced renal inflammation and increased ECM protein production [55,124], while diabetic mice lacking the *miR-146a* gene can have profound increases in proteinuria, glomerular hypertrophy, inflammation, fibrosis, and renal macrophage infiltration compared with those with WT diabetic kidney tissues [126]. Similarly, miR-302d, another protective miRNA, is targeted by TGF-β2 receptors [127]. Increased stimulation of TGF-β receptors, in conditions like chronic hyperglycemia, leads to enhanced suppression of miR-302d [127]. Conversely, upregulation of miR-302d in mice models confers protection against fibrosis, even in the presence of TGF-β receptor stimulation [127]. 

### 5.2. miRNAs in Diabetic Cardiomyopathy (DCM)

Not only can diabetes affect the kidneys, but it can also impact the heart. Diabetic cardiomyopathy is manifested as cardiomyocyte hypertrophy, focal fibrosis, calcium and ATP imbalance, increased ROS formation, and cell death [128]. In the context of miRNAs and DCM, Reddy et al. have previously documented that miRNA-200b/c and miR-429 are upregulated in the aortic vascular smooth muscle cells (VSMCs) of diabetic mice [129]. Notably, in the diabetic VSMCs, the heightened productions of miR-200b and miR-429 are capable of downregulating Zeb1, which subsequently results in the de-repression of several inflammatory genes (i.e., *MCP-1* and *COX-2*). As for other pathogenetic miRNAs, Kishore et al. have demonstrated significant upregulations of miR-155 in the infarcted hearts of db/db mice and the administration of bone marrow-derived progenitor cell therapy augmented cardiac performance by its paracrine effects on cardiac cells; specifically, by inhibiting the pro-fibrotic miR-155 expression levels through hepatocyte growth factor release [130]. Moreover, we and other investigators have reported protective roles of miR-133a in the cardiac hypertrophic response in hyperglycemia [131,132]. We further showed that *miR-133a* transgenic mice are protected against cardiac hypertrophy and fibrosis in diabetes [133]. Our group has also demonstrated that decreased miR-146a expressions are accompanied by overexpression of its target protein, FN, in cardiac tissues of animals with type 1 and 2 diabetes [55]. A more detailed mechanistic study in ECs revealed that p300-mediated histone acetylation regulates the decreased expression levels of miR-146a in hyperglycemia [55]. We have additionally shown that miR-200b prevents both EndMT and increased productions of cardiac ECM proteins [134]. Nevertheless, the different regulatory functionalities of miRNAs highlight the importance of additional exploratory studies that are needed to uncover the roles of other miRNAs in cardiac fibrosis during diabetes.

### 5.3. miRNAs in Diabetic Retinopathy (DR)

In addition to diabetic heart and kidney damage, diabetic retinopathy (DR) is one of the major causes of impaired vision or irreversible vision loss. Multiple biological processes, such as increased angiogenesis, increased permeability, augmented extracellular matrix production, and cell death, are implicated in the progression of this disease. In an array analyses by Kovacs et al., multiple miRNA alterations were also seen in the diabetic retina [135]. Extending these microarray findings, we have carried out several studies in our laboratory with respect to miR-200b at various levels of complexities—from cells to animal and human tissues. In these studies, we have demonstrated that miR-200b can mediate anti-angiogenic and anti-permeability properties by directly targeting VEGF in DR [80]. In accordance with our findings, several investigators have reported a similar inverse relationship between miR-200b and VEGF in DR [136,137]. Of note, the actions of miR-200b may also be mediated through its regulatory role on the histone acetylator, p300 [80]. Using a transgenic mice model with endothelial-specific overexpression of miR-200b, we have further shown that this miRNA regulates EndMT and ECM protein production in multiple organs affected by chronic diabetic complications [40]. Moreover, we have additionally demonstrated that miR-146a, which is regulated by p300, is also responsible for increased ECM protein production in both in vitro and animal models of type 1 and 2 diabetes [55].

## 6. Role of LncRNAs in Chronic Diabetic Complications in Various Organs

Compared with those on miRNAs, fewer studies have focused on lncRNAs with respect to increased ECM protein production and fibrosis in diabetes. Dysregulation of target genes leads to abnormal expressions of lncRNAs that are responsible for cellular defects and disease progression, including carcinogenesis and neurodegeneration [138,139]. *MALAT1*, *lincRNA-p21*, *HOTAIR*, *LSINCT5*, *PTCSC3*, and *H19* are some of the lncRNAs associated with various kinds of cancers [140]. Another lncRNA, *CDKN2B*-AS1 (*ANRIL*), has also been linked to cancer, diabetes, and cardiovascular diseases [141]. Although the lncRNA networks still remain highly elusive in diabetes, several lncRNA-based studies in diabetes-associated complications are beginning to emerge in the hopes of providing novel insights into this complex paradigm (summarized in Table 2).

### 6.1. LncRNAs in Diabetic Nephropathy (DN)

As numerous lncRNAs are being discovered, several analyses are being performed in parallel to characterize the molecular networks involving these unique transcripts. Multiple lncRNAs, such as *PVTI*, *MALAT1*, *GM4419*, *GM5524*, and *ANRIL* [142,143,144,145,146,147,148,149,150,151,152,153,154], have been shown to have versatile roles in the pathogenesis of DN. For example, in DN, TGF-β has been reported to activate Akt by stimulating the expression of miR-216a and miR-217, along with their host lncRNA *RP23*, as well as miR-192 and its host lncRNA, *CJ241444*, resulting in p300 activation [142,145]. These alterations resulted in mesangial cell proliferation and hypertrophy. Another lncRNA-related gene, plasmacytoma variant translocation 1 (*PVT1*), was classified as a candidate gene for end-stage renal disease (ESRD) in type 2 diabetes and was later found to be associated with ESRD in type 1 diabetes as well [143,144]. Upregulated under high glucose conditions, the lncRNA *PVT1* was revealed to be a regulator of the ECM (promotes the expression of FN and type IV collagen), and targeted TGF-β1 and PAI-1 [146]. Strikingly, *PVT1* gene knockdown prevented such high glucose-induced changes [122,146]. Furthermore, the lncRNA *MALAT1* (metastasis associated lung adenocarcinoma transcript 1), originally described in lung adenocarcinoma, is also expressed in the kidneys [147]. *MALAT1* is important in mediating inflammatory reactions in the endothelium and in the kidneys, through which it may possibly influence renal fibrosis [148,149]. In addition to *MALAT1*, the lncRNA *Gm4419*, through its role on p50 (a NF-κB subunit), may also regulate inflammation, fibrosis, and mesangial expansion in DN [150]. Of similar note, Feng et al. have additionally documented that the dysregulated levels of lncRNAs *GM5524* and *GM15645* contribute to podocytes apoptosis and autophagy in DN [151].

Another lncRNA, *NR_033515*, is increased in the serum of DN patients [152]. Elevated levels of *NR_033515* promote mesangial proliferation and EMT, and increase fibrogenesis-related proteins (P38, ASK1, FN, and α-SMA). Gao et al. further document that some of these effects are mediated through miR-743b-5p expressions [152]. A similar phenomenon was also noted in a recent study by Sun et al., where *Erbb4-IR*, a Smad3-dependent lncRNA, promoted renal fibrosis in type 2 DN by suppressing miR-29b [153]. Moreover, the lncRNA antisense non-coding RNA in the *INK4* locus (*ANRIL*) is significantly upregulated in ECs following hyperglycemia [81,154]. We have previously demonstrated that *ANRIL* regulates augmented ECM protein production (FN and Col1α4) in DN and such changes are prevented in the *ANRIL* KO mice [154]. This regulation is at least in part driven by *ANRIL*’s interaction with PRC2 (please see Section 7.1) and the histone acetyltransferase, p300 [81,154]. Similarly, the lncRNA *ASncmtRNA-2* can also regulate diabetes-induced renal fibrosis through TGF-β [155], which suggests that some pathogenetic lncRNAs may exhibit similar functional characteristics in disease progression despite having different sequences. It is of further interest to note that the lncRNA *TUG1*, which is downregulated in the kidneys in diabetes, may additionally control the expression of key fibrosis-related genes such as *collagen*, *FN*, and *TGF-β* [156].

Diabetes can also activate the endoplasmic reticulum (ER)-stress response, which can ultimately lead to the accumulation of unfolded ER proteins and subsequent cellular dysfunction [157]. Intriguingly, the transcription factor CHOP (C/EBP homologous protein) is activated during this stress response [157] and has been shown to regulate a megacluster of miRNAs (~40 miRNAs) and their host lncRNA, *lnc-MGC* [158]. Kato et al. demonstrate that diabetes-induced increases of the miRNA megacluster and *lnc-MGC* were both hindered in the glomeruli of *Chop* KO diabetic mice. Accordingly, compared with WT diabetic mice kidneys, glomerular hypertrophy and the increased expressions of several pro-fibrotic genes (*TGF-β1* and *collagen*) were significantly reduced in the *Chop* KO diabetic mice [158]. Nevertheless, these interesting results allude to the several metabolic abnormalities that are implicated in the biogenesis of miRNAs and lncRNAs during diabetes.

### 6.2. LncRNAs in Diabetic Cardiomyopathy (DCM) and Other Cardiovascular Complications

In the context of cardiovascular disease, lncRNA-based studies in this area are rapidly beginning to emerge. However, presently, lncRNA research using DCM models is limited. Instead, a tremendous amount of research is available for cardiac fibrosis using the myocardial infarction (MI) model. As myocardial fibrosis is quite common in diabetic patients [159], understanding the molecular basis of MI-induced cardiac fibrosis may provide unique insights into the mechanisms implicated in diabetes-associated cardiac fibrosis. Therefore, in this subsection, we will also refer to several studies that have used the MI animal model. 

To begin, in a microarray conducted by Qu et al., the peri-infarct myocardial regions of MI-induced mice revealed nearly 545 lncRNAs (282 downregulated and 263 upregulated) and 209 mRNAs (67 downregulated and 142 upregulated) that were differentially expressed at significant levels [160]. Using stringent cut-off criteria, additional bioinformatic analysis was performed to construct a possible co-expression network of these lncRNAs and mRNAs. Their findings demonstrated that the deregulated expressions of lncRNAs significantly correlated with the expressions of fibrosis-related mRNAs. For example, the lncRNA *NONMMUT022554* was upregulated in MI tissues and positively correlated with six fibrosis-related coding genes (*collagens 1a1*, *1a2*, *3a1*, *4a1*, *5a2*, and *Fn1*). Building on their initial findings, Liang et al. further characterized the underlying mechanisms of the lncRNA *PFL* (profibrotic long non-coding RNA; *NONMMUT022555*) in cardiac fibrosis [161]. In their study, the administration of a *PFL*-specific short hairpin RNA significantly reduced the levels of *PFL* in the hearts of MI mice, and the knockdown also attenuated cardiac fibrosis by decreasing collagen deposition, improving heart function, and reducing the expressions of several fibrosis-related genes at the RNA and protein level [161]. Moreover, in a separate study, Qu et al. characterized the role of another lncRNA known as myocardial infarction associated transcript (*MIAT*). Knockdown of *MIAT* dramatically reduced infarct size and ECM deposition in the cardiac tissues of MI mice [162]. To further confirm their in vivo observations, ex vivo experiments were carried out using neonatal mouse cardiac fibroblasts and the results indicated that *MIAT* knockdown significantly reduced proliferation and collagen content in high serum or angiotensin II-treated cells—abrogating the pro-fibrotic alterations. In addition to *PFL* and *MIAT*, *Wisper* is another lncRNA that plays a central role in cardiac fibrosis and remodeling [163]. As shown by Micheletti and colleagues, the depletion of *Wisper*, using GapmeRs, in post-MI mice dramatically resulted in improved cardiac function, decreased remodeling, and significantly reduced expressions of pertinent ECM and profibrotic protein-coding genes (*Tgfb2*, *Col1a1*, *Col3a1*, *Fn1*, *aSMA*, and *Bcl2*) [163]. Similarly, treatment with *Meg3* GapmeR in mice subjected to transverse aortic constriction also evoked anti-fibrotic responses such as the downregulation of cardiac matrix metalloproteinase-2 (*Mmp2*) and improved diastolic functioning [164].

Using a streptozotocin (STZ)-induced diabetes animal model, recent studies from our laboratory have demonstrated that the lncRNAs *ANRIL* and *MALAT1* have consequential roles in the pathogenesis of DCM. In fact, in the cardiac tissues of *ANRIL* KO diabetic animals, significant reductions in FN, collagen, and VEGF transcripts were observed compared with cardiac tissues from the wild type (WT) diabetic animals [154]. In parallel to our RT-qPCR results, the cardiac tissues from *ANRIL* KO diabetic animals also presented with reduced immunohistochemical staining for FN and collagen, which suggests that *ANRIL* may be capable of regulating ECM protein production in DCM. As for *MALAT1*, our in vivo experiments demonstrated that *Malat1* KO diabetic cardiac tissues had significantly lower levels of inflammatory cytokines (i.e., IL-6, IL-1β, and TNF-α) compared with WT diabetic controls [148]. In addition to the reduction in inflammation, echocardiographic analyses indicated that *Malat1* KO attenuated diabetes-induced cardiac dysfunction. Other investigators have reported similar upregulated patterns of *MALAT1* in cardiac tissues [165,166] and cardioprotective effects following *MALAT1* knockdown during DCM [166]. Although the relationship between *MALAT1* and ECM protein production has not been explored yet in DCM, future studies must examine *MALAT1* and other pertinent lncRNAs using appropriate experimental models for DCM.

### 6.3. lncRNAs in Diabetic Retinopathy (DR)

As for DR, lncRNA expression profiling of retinas from STZ-induced diabetic mice showed alterations of 303 lncRNAs in early DR, of which 214 were downregulated and 89 were upregulated [167]. Among these, *MALAT1*, a conserved lncRNA, was significantly upregulated in an RF/6A cell model of hyperglycemia, in the aqueous humors, and in the fibrovascular membranes of diabetic patients [167]. In accordance with the findings from Yan et al., both in vitro and in vivo results from our laboratory have identified that hyperglycemia induces an upregulation of *MALAT1* in ECs and in the retina, which in turn regulates increased expression of inflammatory mediators, IL-6, and TNF-α, through serum amyloid antigen three (SAA3) activation [149]. When we further examined the functional role of *MALAT1* in DR in vivo, *Malat1* KO dampened the expressions of inflammatory and PRC2 transcripts in the retinal tissues of diabetic mice [168]. Furthermore, our recent study identified for the first time that the vitreous humors of proliferative DR patients had elevated levels of *MALAT1*, which was also associated with increased expressions of inflammatory transcripts (IL-6 and TNF-α). Another study from our laboratory investigated the implications of *ANRIL* on VEGF regulation in DR [81]. *ANRIL* knockdown, using a siRNA-mediated approach, in high glucose-treated human retinal endothelial cells (HRECs) prevented the upregulation of VEGF mRNA and proteins. Similarly, diabetes-induced retinal upregulation of VEGF and elevated retinal microvascular permeability were prevented in the *ANRIL* KO diabetic animals, thereby confirming our in vitro results. Furthermore, RNA immunoprecipitation results from this study also revealed that high glucose exposure promotes a strong physical association between *ANRIL* and two other important epigenetic RNA-binding proteins, p300 and enhancer of zeste homolog 2 (EZH2; a catalytic subunit of PRC2) [81]. Given this finding, along with previous documentations of lncRNAs binding with chromatin remodeling complexes [169], VEGF regulation, as well as increased ECM protein production, may involve *ANRIL*-mediated control of PRC2 components, p300 and miR-200b, as silencing of *ANRIL* impacts the expressions of these molecules [81,154].

To further add to this complex epigenetic paradigm in DR, we have recently looked at the role of the lncRNA *H19* in endothelial–mesenchymal transition (EndMT), which is a process by which ECs lose their endothelial markers and acquire a more mesenchymal phenotype [170]. Despite being downregulated in the retina in diabetes, *H19* was shown to control endothelial–mesenchymal transition (EndMT), independent of miR-200b [170]. In fact, overexpressing *H19* in high glucose-treated HRECs allowed for significant upregulation of endothelial markers (*CD-31* and *VE-CAD*) and drastic downregulation of mesenchymal markers (*FSP-1*, *SM22*, and *α-SMA*), opposite from the trends observed in high glucose controls. These alterations were further confirmed in vivo, in which the diabetic retinal tissues from the *H19* KO animals demonstrated a diabetes-like EndMT phenotype (increased mesenchymal and reduced endothelial markers). Building on our in vitro and in vivo findings, Luminex technology and Western blotting additionally identified that *H19* suppresses glucose-mediated EndMT through regulation of the MAPK-ERK1/2 pathway of TGF-β signalling.

## 7. Relationship of lncRNAs, miRNAs, and Other Epigenetic Mechanisms on Causing Increased ECM Protein Production in Diabetes

**Figure 1:**
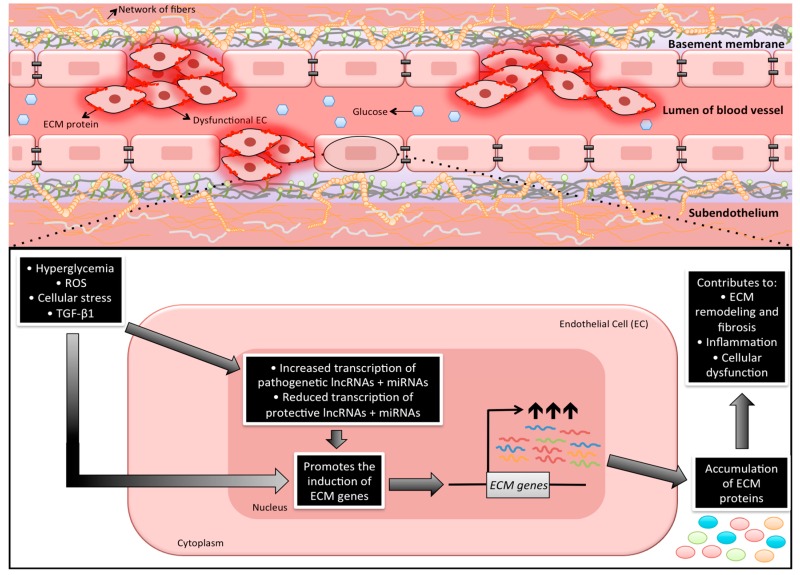
An illustration depicting the potential involvement of long non-coding RNAs (lncRNAs) and microRNAs (miRNAs) in the regulation of extracellular matrix proteins in endothelial cells during diabetes. As endothelial cells (ECs) are the first cell types to be exposed to systemic hyperglycemia, increased intracellular glucose triggers a number of metabolic disturbances. Among these disturbances, chronic hyperglycemia can cause the endothelium to lose its inherent physiological properties. Because these changes work primarily through the transcriptional machinery of the cell, recent research is beginning to demonstrate that non-coding RNAs are implicated in this complex pathogenetic process. In fact, the aberrant production of extracellular matrix (ECM) proteins in diabetes has been linked to many miRNAs and lncRNAs, which suggests that these non-coding RNAs are key epigenetic regulators in such a synthetic process. Additional notes: Cells denoted as “dysfunctional EC” in the image above are showcasing the abnormal ECs that have heightened production and deposition of ECM proteins. As well, the endothelial basement membrane is comprised of a complex network of fibers (includes collagen IV and laminin) that crosslink with many other proteins (i.e., perlecans). Endothelial cells are connected to this network by the presence of integrins (the orange molecules in the diagram). Of note, it has been documented that early hyperglycemia can provoke basement membrane thickening, which can have many detrimental consequences, such as the inability to prevent vascular permeability [178].

As noted earlier, it is generally accepted that ECs are primary targets of glucose-induced tissue damage [6,7,11]. In ECs, multiple glucose-induced biochemical alterations coverage onto the cell nucleus and changes in gene transcription ultimately produce increased amounts of cellular macromolecules. Because of this dynamic process, it is important to have a better understanding of such cellular biosynthetic processes. Studies have been previously carried out examining the specific genetic abnormalities as causative factors for the development of chronic diabetic complications. However, such studies have never been able to provide definitive answers [171]. On the other hand, metabolic memory phenomenon and legacy effects of diabetes are well established [172,173,174]. Among these, the roles of lncRNAs and miRNAs are relatively understudied. Nevertheless, current literature suggests that these unique ncRNAs play extensive roles in gene regulation and are able to exert various functions in a wide variety of cellular contexts. On the basis of our discussion above, multiple lncRNAs and miRNAs can also be involved in the increased production of ECM proteins (Figure 1)—some of these may be tissue-specific. Regardless, an increasing body of evidence points to the notion that ncRNAs are capable of interacting with each other and can be further co-regulated during various pathological conditions [175,176,177]. 

Specifically, in the context of chronic diabetic complications, several pathways can regulate the targets under investigation. We have previously shown that VEGF can be regulated by miR-200b, p300, and PRC2 [40,80,179]. Our recent research has revealed another layer of regulation for VEGF in this pathway. We have demonstrated that *ANRIL*, through its interaction with miR-200b, p300, and PRC2, can mediate the production of VEGF and ECM proteins [81,154]. These findings for the first time demonstrated such a role for ANRIL in the context of diabetic complications. However, in other systems, ANRIL has been shown to specifically bind to PRC2 proteins and subsequently regulate histone modifications [105]. As for histone acetylation, we have previously demonstrated that p300 regulates a large number of transcripts that are induced by glucose [49]. Hence, p300’s regulation by ANRIL may, at least in part, provide an explanation for the regulation of multiple seemingly diverse transcripts that are altered by glucose.

Nevertheless, although it may be possible that the unique interactions between PRC2, p300, miRNAs, and lncRNAs may govern ECM remodeling during diabetes, future studies should explore the interactions between these ncRNAs and important epigenetic mediator proteins in more detail.

### 7.1. Histone Methylation: PRC2 and lncRNAs

Histone methylation is closely involved in gene expression through lncRNAs [180,181]. Depending on which amino acid residue in the histone becomes methylated, the result can be activation or repression of gene expression [180,181,182]. Chromatin-modifying enzymes, such as methyltransferases, are often comprised of adaptor proteins that help maintain the stability and genomic functionality of the complex [183,184]. For example, supressor of Zeste 12 (SUZ12) and embryo ectoderm development (EED) are critical adaptor proteins that interact with enhancer of the zeste homolog 2 (EZH2) to form polycomb repressive complex 2 (PRC2), the major methyltransferase complex for H3K27 methylation. EED and SUZ12 allow for recruitment to specific areas of the genome [184], which can also function through lncRNAs [185]. LncRNAs are able to guide chromatin-modifying enzymes to specific genomic regions through the unique structural domains present on the lncRNA [92,98,185]. Similarly, lncRNAs can act as molecular scaffolds to increase interactions between transcription factors and other chromatin modification enzymes [186]. Of note, H3K27me3 has also been linked to miRNA regulation [187]. As evidenced by several studies, the binding specificity of PRC2 to different regions of the genome is mediated by direct protein-DNA interactions and by recruitment through lncRNAs, such as *ANRIL*, which function as molecular flags to direct PRC2 [105,180,181,182,183,184,185,186,187]. However, further detailed discussion of these additional epigenetic phenomena and their interactions are beyond the scope of this review.

## 8. Concluding Remarks

It is important to note that nature has provided us with a multilayered control for gene expression. An example of direct relevance to the current discussion is that we have shown miR-200b, directly or through p300, to regulate EndMT and increased ECM protein productions in diabetes. Adding to this layer of complexity, we have recently shown that the lncRNA *H19* is also capable of regulating such processes. Furthermore, aside from miRNAs and lncRNAs, other novel ncRNA molecules are continuously being discovered as genomic technologies improve, which will greatly enhance our understanding of disease pathogenesis. For instance, powerful sequencing tools are beginning to identify several emerging players in diabetes research: circular RNAs [188], piwi-interacting RNAs [189], and snoRNAs [190]. Hence, a symphony involving acetylation, deacetylation, ncRNAs, DNA, and/or histone methylation, and possibly other yet-unidentified mechanisms, may regulate gene expression and cellular phenotypic alterations that lead to increased ECM protein production in chronic diabetic complications. Identifying the conductor of this orchestra may provide us with an effective drug target. Paradoxically, destabilizing such a conductor may also come with a price, as the majority of players are integrated in a large coordinated network of signalling pathways that ultimately regulate physiological cellular functions. Alternatively, targeting a specific player of this orchestra using RNA-based therapeutics may provide us with an opportunity to block a disease and/or cell-specific mechanism. However, this notion needs to be established by well-designed long-term studies. Nevertheless, this review summarized the key mechanisms involving ncRNAs, specifically miRNAs and lncRNAs (shown in Table 1 and Table 2), with respect to increased ECM protein production implicated in fibrosis during diabetes. Although direct evidence for the mechanisms of ncRNAs is still limited in the context of diabetes-associated fibrosis, emerging studies are beginning to provide better insights into the complexity of the epigenome, necessitating the need for additional research behind ncRNAs and epigenetic interactions. We hope that our review will allow for critical discussions and further experimentation. 

## Figures and Tables

**Table 1:** Examples of certain micro RNAs (miRNAs) implicated in diabetes.

miRNA	Cell/Tissue Type(s)	Reported Function(s)	Disease Model(s)
miR-192 *	• Mouse mesangial cells • Human proximal tubule cells and kidneys • Rat tubular epithelial cells • Human podocytes	• Elevated expressions associated with increased *Col1a2* expressions; targets SIP1 [108] • Loss of miR-192 expression is associated with increased fibrosis and decreased estimated GFR [111] • Decreased in the diabetic kidney, targets ZEB2, and does not affect extracellular matrix (ECM) protein expressions [112] • Increased in high glucose and diabetic conditions [109,110,142] • Can regulate other transcription factors and miRNAs [109,110]	• DN [108,109,110,111,112,142]
miR-215	• Proximal tubular cells • Rat mesangial cells • Human podocytes	• Decreased in the diabetic kidney • Ectopic expression of miR-215 increases E-cadherin levels by repressing ZEB2 translation	• DN [112]
miR-377	• Human and mouse mesangial cells • Mice kidney tissues	• Up-regulated in hyperglycemic and diabetic conditions • Can indirectly lead to increased fibronectin protein production	• DN [113]
miR-21	• Rat mesangial cells • Mice kidney tissues • Rat tubular epithelial cells	• Expression is increased in DN and can enhance the production of high glucose-induced fibrotic and inflammatory markers	• DN [125]
miR-29*	• Human podocytes • Mouse mesangial cells • Mice kidney tissues • Mouse embryonic fibroblasts and tubular epithelial cells • Mice kidney glomeruli, endothelial cells, and podocytes	• Low levels in early DN and fibrosis and can target collagens I and IV [116] • Lost with progressive renal fibrosis, can reduce collagens I and III, and interact with Smad3 [117] • miR-29c is increased in DN, induces cell apoptosis, and increases ECM protein accumulation [118]	• DN [116,117,118]
miR-let-7b	• Rat proximal tubular epithelial cells • Mice kidney tissues	• Is reduced in both diabetic and non-diabetic renal fibrosis and can regulate the expression of several ECM genes	• DN [114]
miR-93	• Renal microvascular endothelial cells • Mouse podocytes • Mice kidney tissues	• High glucose and diabetic conditions decrease miR-93 expressions • Can target VEGF and negatively regulate it	• DN [115]
miR-200 *	• Human retinal endothelial cells • Mice and rat retinal tissues • Mice kidney tissues • Mouse mesangial cells • Mouse heart endothelial cells, vascular smooth muscle cells, and cardiac tissues	• miR-200b is reduced under hyperglycemic and diabetic conditions [40,179]; can target VEGF [80,179] • Inhibition of miR-200a-3p can provoke renal fibrosis in DN [121] • Increased levels of miR-200b/c detected in diabetic mouse glomeruli; involved in glomerular mesangial hypertrophy [110,120] • miR-200b overexpression shown to prevent diabetes-induced changes in heart structure and function and reduce EndMT markers [134] • miR-200b shown to have a protective role in the diabetic retina [136,137] • Diabetic VSMCs exhibit increased miR-200 levels, which can contribute to inflammation [129]	• DR [40,80,136,137] • DN [110,120,121,179] • DCM [129,134]
miR-146a	• Rat and mice retinal tissues • Human umbilical vein endothelial cells • Mice kidney tissues	• miR-146a was shown to be reduced in diabetic tissues; miR-146a mimics can decrease FN expression [55] • *miR-146a* knockout exacerbates diabetes-induced inflammation and fibrosis in mice kidney tissues [126] • miR-146a mimics can prevent the increased expressions of ECM proteins and inflammatory markers in diabetic tissues [124]	• DR [55] • DCM [55] • DN [124,126]
miR-1207-5b	• Human renal proximal tubule epithelial cells, podocytes, and mesangial cells	• Hyperglycemia shown to increase miR-1207-5b levels, which contributes to ECM accumulation in the kidney • Knockdown can decrease levels of TGF-β1, FN1, and PAI-1	• DN [122]
miR-302d	• Mice kidney tissues • Human mesangial cells, proximal tubular epithelial cells, and HEK-293T	• Capable of attenuating TGF-β-induced fibronectin, thrombospondin, vimentin, and N-cadherin expressions • Can regulate TGF-β -induced EMT	• DN [127]
miR-216a	• Primary mouse mesangial cells (MMCs)	• Upregulated by TGF-β in MMCs • Also increased in isolated renal glomeruli from type 1 and type 2 diabetic mice • Inhibiting miR-216a in MMCs reverses the effects of TGF-β on Pten and P-Akt levels	• DN [145]
miR-217	• Primary mouse mesangial cells	• Upregulated by TGF-β in MMCs • Also increased in diabetic mice kidneys • Along with miR-216a, miR-217 mimics can induce hypertrophy in MMCs	• DN [145]
miR-133a	• Mice cardiac tissues • Neonatal rat myocytes	• Downregulated in diabetic cardiomyopathy [131,132,133] • Mediates glucose-induced cardiomyocytes hypertrophy [131,132,133] • Cardiac-specific overexpression of miR-133a can significantly decrease cardiac fibrosis [133]	• DCM [131,132,133]
miR-155	• Mouse cardiac fibroblasts • Mouse bone marrow progenitor cells (BMPCs)	• Increased expression of miR-155 in MI mice • Transplantation of BMPCs in MI mice can decrease miR-155 expressions and in association, show decreased cardiac fibrosis expressions	• DCM [130]

* indicates different reported effects of the miRNA in literature; DN = diabetic nephropathy; DR = diabetic retinopathy; DCM = diabetic cardiomyopathy; MI = myocardial infarction; ECM = extracellular matrix; VSMCs = vascular smooth muscle cells; VEGF = vascular endothelial growth factor; SIP1 = Smad-interacting protein 1; ZEB2 = Zinc Finger E-box Binding Homeobox 2; GFR = glomerular filtration rate; EMT = epithelial–mesenchymal transition; EndMT = endothelial–mesenchymal transition.

**Table 2:** LncRNAs currently implicated in diabetes and other disease-associated models.

lncRNA	Cell/Tissue Type(s)	Reported Function(s)	Disease Model
***PVT1***	• Humanmesangial cells	• Upregulated by glucose treatment in mesangial cells • *PVT1* knockdown can significantly reduce the levels of major ECM proteins (FN and COL4A1)	• DN [146]
***MALAT1***	• Mice kidney tissues • Mouse podocytes • Mice and rat cardiac tissues • HRECs • Mice retinal tissues • RF/6A cells • Aqueous and vitreous humors	• *MALAT1* levels are increased in the kidney cortices of STZ-induced diabetic mice [147] • *MALAT1* regulates diabetes-induced inflammatory gene expressions in the heart and kidneys [148,149] • *MALAT1* is upregulated in HG-treated RF/6A cells, aqueous humor samples and in fibrovascular membranes of diabetic patients [167] • *MALAT1* has a pathogenetic role in the heart [165,166] • *MALAT1* can regulate inflammation through its association with other epigenetic mechanisms in DR [168]	• DN [147,148,149] • DR [167,168] • DCM [148,165,166]
***GM4419***	• Mouse mesangial cells (MMCs)	• Upregulated in MMCs following high glucose culture • Knockdown of *GM4419* inhibits the glucose-induced expressions of pro-inflammatory cytokines and renal fibrosis markers • *GM4419* can regulate NF-κB signalling	• DN [150]
***GM5524***	• Mice kidney tissues • Mouse podocytes	• Expressions are significantly upregulated in DN • May regulate podocytes apoptosis and autophagy during DN	• DN [151]
***GM15645***	• Mice kidney tissues • Mouse podocytes	• Downregulated in DN • Similar to *GM5524*, *GM15645* may also regulate podocytes apoptosis and autophagy during DN	• DN [151]
***ANRIL***	• Human retinal endothelial cells (HRECs) • Mice retinal tissues • Mice kidney and cardiac tissues	• High glucose and diabetic conditions upregulate *ANRIL* expressions [81,154] • *ANRIL* can regulate VEGF and ECM expressions through several epigenetic mechanisms (i.e., p300 and PRC2) [81,154]	• DN [154] • DR [81] • DCM [154]
***NR_033515***	• Human blood • HEK293-T • MMCs	• Significantly increased in the serum of DN patients • Overexpression of *NR_033515* can accelerate TGF-β1-induced EMT • Promotes cell proliferation and fibrogenesis in high glucose conditions	• DN [152]
***Erbb4-IR***	• Mice kidney tissues • Mouse embryonic fibroblasts • MMCs • Mouse tubular epithelial cells	• Significantly upregulated in the kidneys of diabetic mice • A Smad3-dependent lncRNA that promotes renal fibrosis in type 2 DN • Can negatively regulate miR-29b • Kidney-specific silencing of *Erbb4-IR* shown to prevent renal injury in diabetic mice	• DN [153]
***ASncmtRNA-2***	• Mice kidney tissues • Human mesangial cells	• Expressions are significantly heightened in diabetic mice kidneys and mesangial cells treated with high glucose • May promote glomerular fibrosis in DN	• DN [155]
***Tug1***	• Mice kidney tissues • MMCs	• Suppresses the proliferation of mesangial cells and decreases the expression of ECM-associated proteins in DN • Functions as an endogenous sponge of miR-377, which directly targets PPARγ	• DN [156]
***NONMMUT022554***	• Mice cardiac tissues	• Upregulated in cardiac fibrosis and positively correlated with 6 upregulated genes involved in ECM–receptor interactions and the PI3K–Akt signalling pathway	• Cardiac fibrosis/MI [160]
***PFL (NONMMUT022555)***	• Mice cardiac tissues • Mice cardiac fibroblasts and cardiomyocytes	• Upregulated in the hearts of MI mice • Knockdown of *PFL* can attenuate cardiac interstitial fibrosis and improve cardiac function • Overexpression of *PFL* promotes proliferation, fibroblast-myofibroblast transition, and mice cardiac fibroblasts • Acts as a competitive endogenous RNA of let-7d	• Cardiac fibrosis/MI [161]
***MIAT***	• Mice cardiac tissues • Mouse cardiac fibroblasts	• Significantly upregulated in the infarcted myocardium of mice • Knockdown of *MIAT* reduces cardiac fibrosis and improves cardiac function • Functions as a sponge of miR-24 in cardiac fibroblasts	• Cardiac fibrosis/MI [162]
***Wisper***	• Mice and human cardiac tissues • Mouse cardiac fibroblasts and cardiomyocytes • Human fibroblasts	• Expression of *Wisper* is strongly correlated with cardiac fibrosis in both animal and human heart tissues • *Wisper* knockdown affects cardiac fibroblast survival, migration, and proliferation • In vivo depletion of *Wisper* inhibits cardiac fibrosis and improves function	• Cardiac fibrosis/MI [163]
***Meg3***	• Mice cardiac tissues • Mouse cardiac fibroblasts	• Strongly expressed in adult cardiac fibroblasts • Regulates the production of MMP-2 in vitro • In vivo inhibition of *Meg3* after transverse aortic constriction decreases cardiac fibrosis and improves diastolic function	• Cardiac fibrosis [164]
***H19***	• HRECs • Mice retinal tissues • Human vitreous humors	• Downregulated in HG-treated endothelial cells and in the vitreous humors of diabetic patients • Capable of regulating EndMT in vitro and in vivo	• DR [170]
***Lnc-MGC***	• Mice kidney tissues • Mouse and human mesangial cells • Human renal biopsies	• Elevated levels of *lnc-MGC* present in the kidneys during diabetes • Host to a megacluster of miRNAs • CHOP (an ER-stress transcription factor) regulates *lnc-MGC* expressions • Inhibition of *lnc-MGC* results in reduced cluster miRNAs, ECM accumulation, and glomerular hypertrophy	• DN [158]

DN = diabetic nephropathy; DR = diabetic retinopathy; DCM = diabetic cardiomyopathy; MI = myocardial infarction; ECM = extracellular matrix; STZ = streptozotocin; PPARγ = peroxisome proliferator-activated receptor gamma; MMP-2 = matrix metalloproteinase-2; EMT = epithelial–mesenchymal transition; EndMT = endothelial–mesenchymal transition; HG = high glucose.
